# Hypoxia Modulates Radiosensitivity and Response to Different Radiation Qualities in A549 Non-Small Cell Lung Cancer (NSCLC) Cells

**DOI:** 10.3390/ijms25021010

**Published:** 2024-01-13

**Authors:** Hasan Nisar, Frederik M. Labonté, Marie Denise Roggan, Claudia Schmitz, François Chevalier, Bikash Konda, Sebastian Diegeler, Christa Baumstark-Khan, Christine E. Hellweg

**Affiliations:** 1 Department of Radiation Biology, Institute of Aerospace Medicine, German Aerospace Center (DLR), 51147 Cologne, Germany; hasanisar@pieas.edu.pk (H.N.); flabonte@smail.uni-koeln.de (F.M.L.); denise.roggan@dzne.de (M.D.R.); claudia.schmitz@dlr.de (C.S.); bikash.konda@dlr.de (B.K.); sebastian.diegeler@utsouthwestern.edu (S.D.); christa.baumstark-khan@dlr.de (C.B.-K.); 2 Department of Medical Sciences, Pakistan Institute of Engineering and Applied Sciences (PIEAS), Islamabad 44000, Pakistan; 3 Center for Molecular Medicine Cologne (CMMC), University of Cologne, 50931 Cologne, Germany; 4 German Center for Neurodegenerative Diseases (DZNE), 53127 Bonn, Germany; 5 UMR6252 CIMAP, CEA-CNRS-ENSICAEN-University of Caen Normandy, 14000 Caen, France; francois.chevalier@ganil.fr; 6 Department of Radiation Oncology, UT Southwestern Medical Center, Dallas, TX 75390, USA

**Keywords:** NSCLC, cellular hypoxia, radiosensitivity, high-LET radiation, cell cycle response, colony forming ability, PI3K responsive genes

## Abstract

Hypoxia-induced radioresistance reduces the efficacy of radiotherapy for solid malignancies, including non-small cell lung cancer (NSCLC). Cellular hypoxia can confer radioresistance through cellular and tumor micro-environment adaptations. Until recently, studies evaluating radioresistance secondary to hypoxia were designed to maintain cellular hypoxia only before and during irradiation, while any handling of post-irradiated cells was carried out in standard oxic conditions due to the unavailability of hypoxia workstations. This limited the possibility of simulating in vivo or clinical conditions in vitro. The presence of molecular oxygen is more important for the radiotoxicity of low-linear energy transfer (LET) radiation (e.g., X-rays) than that of high-LET carbon (^12^C) ions. The mechanisms responsible for ^12^C ions’ potential to overcome hypoxia-induced radioresistance are currently not fully understood. Therefore, the radioresistance of hypoxic A549 NSCLC cells following exposure to X-rays or ^12^C ions was investigated along with cell cycle progression and gene expression by maintaining hypoxia before, during and after irradiation. A549 cells were incubated under normoxia (20% O_2_) or hypoxia (1% O_2_) for 48 h and then irradiated with X-rays (200 kV) or ^12^C ions (35 MeV/n, LET ~75 keV/µm). Cell survival was evaluated using colony-forming ability (CFA) assays immediately or 24 h after irradiation (late plating). DNA double-strand breaks (DSBs) were analyzed using γH2AX immunofluorescence microscopy. Cell cycle progression was determined by flow cytometry of 4′,6-diamidino-2-phenylindole-stained cells. The global transcription profile post-irradiation was evaluated by RNA sequencing. When hypoxia was maintained before, during and after irradiation, hypoxia-induced radioresistance was observed only in late plating CFA experiments. The killing efficiency of ^12^C ions was much higher than that of X-rays. Cell survival under hypoxia was affected more strongly by the timepoint of plating in the case of X-rays compared to ^12^C ions. Cell cycle arrest following irradiation under hypoxia was less pronounced but more prolonged. DSB induction and resolution following irradiation were not significantly different under normoxia and hypoxia. Gene expression response to irradiation primarily comprised cell cycle regulation for both radiation qualities and oxygen conditions. Several PI3K target genes involved in cell migration and cell motility were differentially upregulated in hypoxic cells. Hypoxia-induced radioresistance may be linked to altered cell cycle response to irradiation and PI3K-mediated changes in cell motility and migration in A549 cells rather than less DNA damage or faster repair.

## 1. Introduction

Radiotherapy is used in half of all patients with solid malignancies, including non-small cell lung cancer (NSCLC). Hypoxia is a well-known cause of radioresistance in tumor cells in vitro and in vivo [1,2]. Cellular hypoxia is demonstrable in up to 80% of NSCLC tumors. Of all lung cancers, 85% are NSCLC; hypoxia-induced radioresistance has been associated with poor prognosis in NSCLC in at least three meta-analyses [3,4]. Clinical trials focusing on hypoxia reversal and radiation dose escalation in solid malignancies, including NSCLC, have met only limited success [3], highlighting the need for greater understanding of cellular pathways and genes that constitute cellular responses to hypoxia. A549 cells are well characterized as a human NSCLC cell line and widely used for studying NSCLC [5].

Radio-resistance becomes measurable at oxygen partial pressures ≤10 mm of Hg (~1% concentration) and reaches a maximum under anoxic conditions [6]. It is in part ascribed to the “Oxygen Fixation Hypothesis”, postulating that the presence of molecular oxygen during irradiation sensitizes cells to ionizing radiation (IR) by ensuring more sustainable production of reactive oxygen species (ROS) that damage DNA [7]. This oxygen effect is influenced by linear energy transfer (LET), an index of radiation quality defined as the dose deposited per unit length of matter. Low-LET IR, such as X-rays, is highly dependent on ROS production for cytotoxicity, whereas high-LET IR, such as heavy nuclei, is less dependent on ROS by directly damaging nuclear DNA in cells. Therefore, cells exposed to X-rays exhibit greater hypoxia-induced radioresistance compared to cells irradiated with high-LET ^12^C ions, while the severity of this effect depends on LET and the extent of hypoxia [8]. ^12^C ions are used in an increasing number of NSCLC clinical trials because they allow more precise dose deposition in the tumor and have higher relative biological effectiveness (RBE) for cell killing compared to X-rays under standard normoxic conditions [9,10,11].

Cellular hypoxia of ≤1% O_2_ can also induce radioresistance through cellular adaptations that affect tumor cell proliferation, energy metabolism, pluripotency, migration and invasion potential, as well as apoptotic, immunologic and inflammatory cellular responses [12,13,14]. The cytotoxicity of both low- and high-LET radiation may be modulated by such hypoxia-mediated adaptations in the DNA damage response (DDR).

The DDR is highly dependent on p53-mediated cell cycle regulation, resulting in activation of cell cycle checkpoints to buy time for repair of damaged DNA. With the additional stress of hypoxia, p53 may also induce apoptosis and autophagy [15]. An obvious adaptation to hypoxia is the slowing of the cell cycle, resulting in the redistribution of cells into the G1 phase, mainly due to hypoxia-mediated mid-G1 arrest [16,17,18] allowing cells time for DNA repair. The final effect would depend on hypoxia’s severity and duration, as well as the specific cell line [18,19]. Under normal oxygenation conditions (normoxia), high-LET radiation produced a stronger G2/M arrest in human hepatoma cells than did low-LET X-rays [20]. However, the impact of radiation quality (high- vs. low-LET) on the backdrop of hypoxia is less well investigated.

Classical radiobiological studies on the effects of hypoxia were usually performed by culturing cells in specialized incubators with regulated oxygen concentration, and irradiation was performed in airtight containers or specialized chambers maintaining a hypoxic environment during IR exposure. However, cell handling after irradiation was performed under normoxia, introducing the effect of reoxygenation after irradiation [21,22,23]. This is in contrast to clinical settings where IR exposure during fractionated radiotherapy generally results in further reduction of tumor perfusion worsening hypoxia and this has been demonstrated multiple times in NSCLC and other tumors [24,25,26,27]. In this work, the effect of prolonged hypoxia continuing even after irradiation was investigated using a combined hypoxia incubator and workstation that allows the uninterrupted handling of cells under hypoxia.

Our study aimed to understand radioresistance and the underlying mechanisms in such continuously hypoxic A549 NSCLC cells following high- and low-LET irradiation. Radiation response of NSCLC cells (A549) under prolonged (48 h) moderate hypoxia (1% O_2_) compared to normoxia was investigated after X-rays and carbon ion irradiation. Radioresistance was determined based on cell survival in terms of reproductive integrity. Cell cycle distribution, DNA repair, and differential gene expression were studied to obtain a complete picture of cellular radiation response under prolonged hypoxia. Cellular sensitivity and response were found to be strongly affected by these prolonged hypoxic conditions in an LET-dependent manner.

## 2. Results

### 2.1. A549 Lung Cancer Reproductive Integrity Depends on Oxygenation Status after X-rays but Not after Carbon Ion Exposure

The plating efficiency of A549 cells under normoxia and hypoxia without irradiation was 35% ± 3% and 25% ± 2% (n = 3), respectively, showing that the cells could grow into colonies at 1% O_2_.

**Figure 1:**
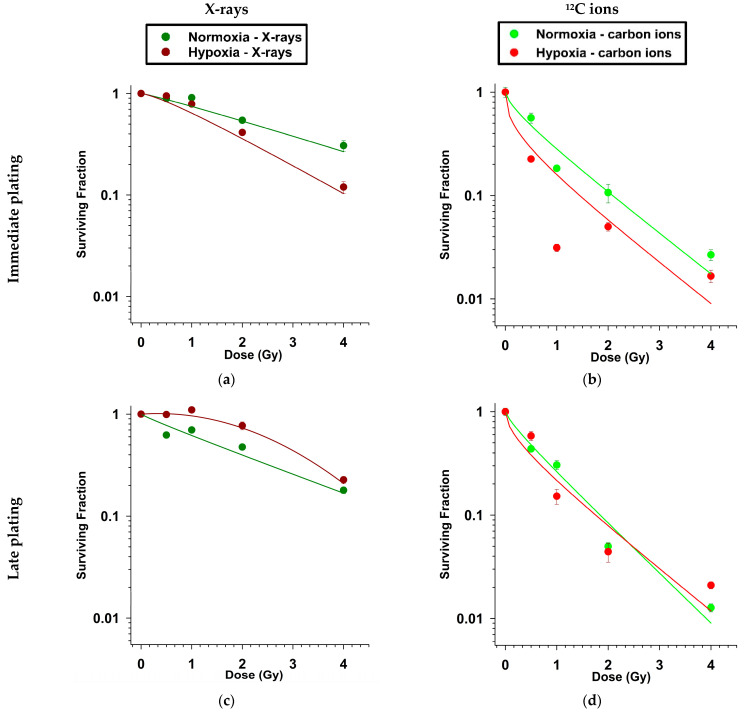
Survival of clonogenic A549 cells after X-ray exposure (**a**,**c**) or carbon ion exposure (**b**,**d**) under normoxia and hypoxia. Following irradiation, cells were either seeded immediately for colonies (immediate plating; (**a**,**b**)) or underwent only a medium change and seeded for colonies after 24 h (late plating; (**c**,**d**)). N = 3. Error bars represent SE.

The radiotoxicity of ^12^C ions compared to X-rays, as quantified by RBE, was greater regardless of oxygenation status (Table 1). Hypoxia-induced radioresistance was observed in late plating (LP) experiments following both X-rays and ^12^C ion exposure as quantified by OER (Table 1). In the case of immediate plating (IP) experiments, D_0_ values indicated lower cell survival under hypoxia compared to normoxia, especially after X-ray exposure (Figure 1, Table 1). The type of plating (IP vs. LP) dramatically reduced D_0_ for hypoxic cells following X-ray exposure but not so after ^12^C ion irradiation.

### 2.2. Cell Cycle Arrest Is Prolonged after Carbon Ion Exposure while Hypoxia Reduces Its Severity for Both Radiation Qualities

After the initial 48 h incubation under hypoxia or normoxia, the average G1 phase population followed over 24 h in the unirradiated samples was significantly greater in hypoxic compared to normoxic A549 cells, while the average G2 population was significantly lower in these cells (Table 2).

**Figure 2:**
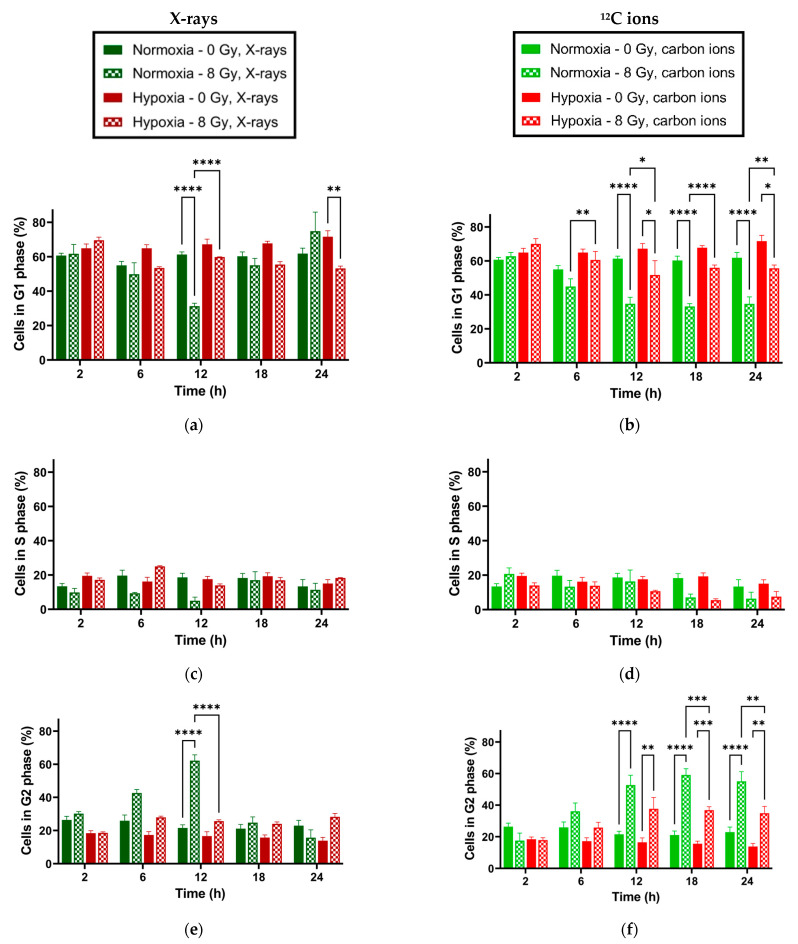
Distribution of A549 cells in cell cycle phases G1 (**a**,**b**), S (**c**,**d**), and G2 (**e**,**f**) at different time points following irradiation with X-rays (**a**,**c**,**e**) or ^12^C ions (**b**,**d**,**f**). Before irradiation, the cells were incubated for 48 h under normoxia or hypoxia (1% O_2_). Significant differences in mean cell populations are represented with asterisks; *: *p* < 0.05; **: *p* < 0.01; ***: *p* < 0.001; ****: *p* < 0.0001; n = 3. Error bars represent SE.

Following X-ray exposure, normoxic cells showed a transient decrease in the G1 phase population concurrent with an increase in G2 cells (Figure 2a,c), indicative of G2 arrest. This response was significant only 12 h post-irradiation. In hypoxic cells, a similar transient response occurred with a significantly decreased G1 population after 24 h but the increase in G2 cells was nonsignificant.

After carbon ion exposure, both normoxic and hypoxic cells showed permanent G2 arrest starting 12 h after irradiation (Figure 2d,f). Compared to X-rays, carbon ion-induced cell cycle response started earlier and remained longer. As with X-rays, the population of cells in G2 under normoxia compared to hypoxia was greater after exposure to carbon ions.

This indicates that the overall cell cycle response of hypoxic A549 to radiation exposure is weaker than the normoxic cell response and, in the case of X-ray exposure, is induced more slowly.

### 2.3. The Number of γH2AX Foci Depends on Radiation Quality but Not on Oxygenation Status

**Figure 3:**
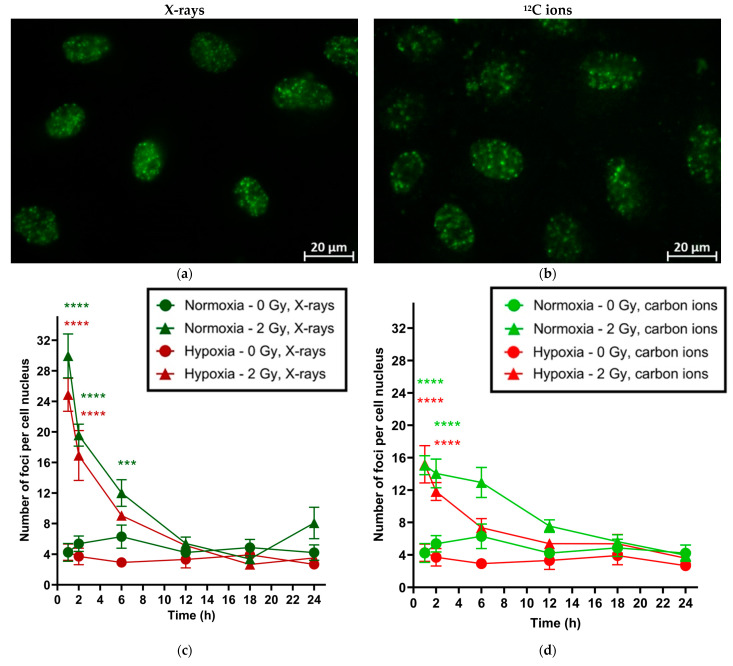
γH2AX foci induction and repair over time in A549 cells under normoxia (20% O_2_) and hypoxia (1% O_2_) following exposure to X-rays or carbon ions. Before irradiation, cells were incubated for 48 h under normoxia or hypoxia (1% O_2_). Exemplary images of A549 cells 1 h after exposure to X-rays (**a**) and ^12^C ions (**b**). Repair kinetics after exposure to X-rays (**c**) and ^12^C ions (**d**). Difference between irradiated and unirradiated samples for both normoxia and hypoxia: *** *p* < 0.001, **** *p* < 0.0001.

γH2AX foci were counted in the A549 cell nuclei periodically (1, 2, 6, 12, 18 and 24 h) over the next 24 h following irradiation with 2 Gy of X-rays or ^12^C ions (Figure 3). Cells were incubated for 48 h under normoxia or hypoxia before irradiation. The average number of γH2AX foci in the cell nuclei of the unirradiated controls was not significantly different under normoxia or hypoxia. There was no significant difference in the initial number of foci (1 h after irradiation) between normoxia and hypoxia following irradiation. The same physical dose of X-rays produced a greater number of foci than ^12^C ions regardless of oxygenation status. Most foci resolved within the first 6 h after irradiation; no statistically significant difference in foci’ resolution kinetics was observed between normoxic and hypoxic cells.

### 2.4. Hypoxic Modulation of Gene Expression Response Differs after X-rays Compared to Carbon Ion Exposure

Gene expression in A549 cells was studied under normoxia and hypoxia 4 h after irradiation with 8 Gy of X-rays and ^12^C ions to correlate gene expression findings with cell cycle changes (Table A1), as well as to identify potential mechanisms behind enhancement of cell survival under hypoxia after exposure to X-rays in the LP CFAs.

**Figure 4:**
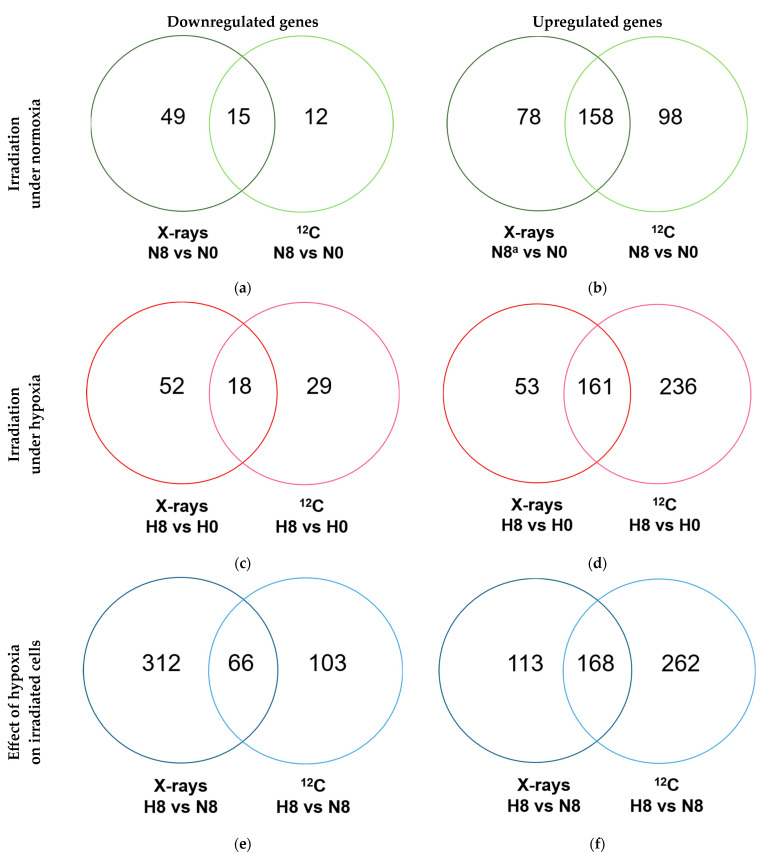
Differentially expressed genes (DEGs) following X-rays and ^12^C ion exposure under normoxia and hypoxia. Venn diagrams of down- (**a**,**c**,**e**) and upregulated (**b**,**d**,**f**) genes 4 h after radiation exposure (8 Gy), following a 48 h preincubation under normoxia (**a**,**b**) or hypoxia (1% O_2_) (**c**,**d**). The number of DEGs for the comparison of hypoxic and normoxic irradiated cells is shown in (**e**,**f**).

Several differentially expressed genes (DEGs) regulated in response to X-rays and ^12^C ion exposure compared to unirradiated controls overlapped, but the majority of regulated DEGs were exclusive to irradiation with either X-rays or ^12^C ions regardless of oxygenation status (Figure 4a–d) and the transcriptional response in hypoxic irradiated cells compared to normoxic irradiated controls showed the same trend (Figure 4e,f).

#### 2.4.1. Irradiation Initiates a Cell Cycle Response That Is Only Slightly Influenced by Radiation Quality and Oxygenation Status

**Figure 5:**
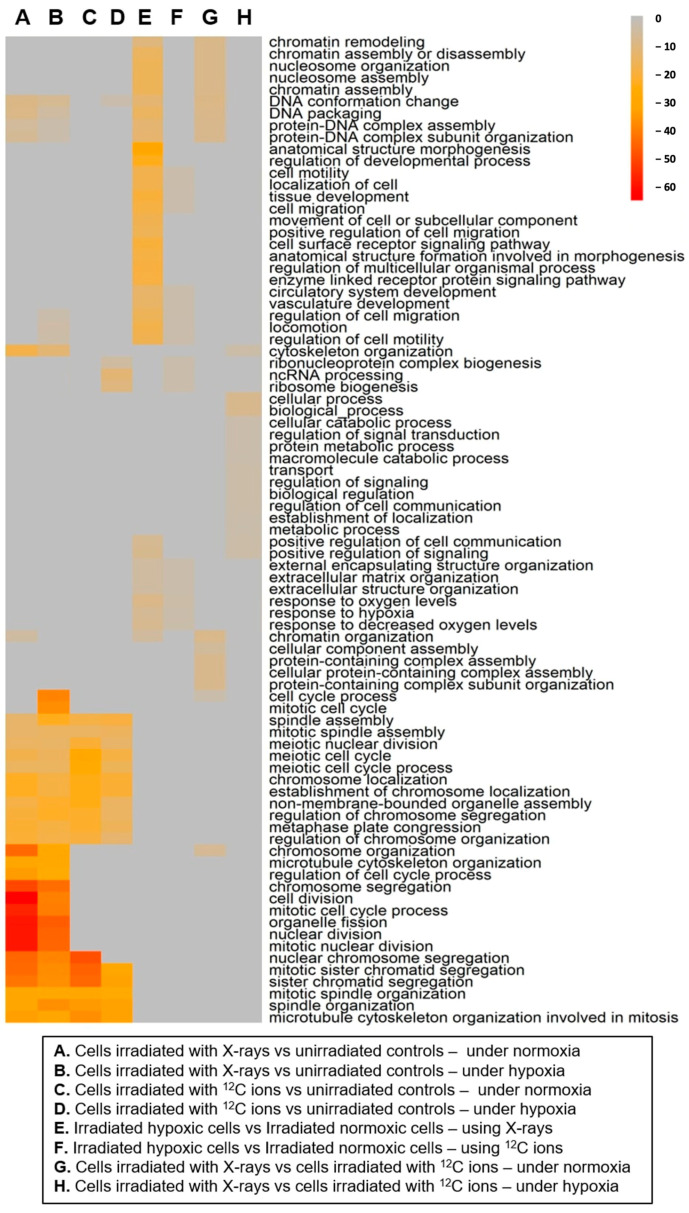
GO terms of differentially expressed genes in A549 cells 4 h after X-rays and ^12^C ion exposure (8 Gy) under normoxia and hypoxia, determined by RNA sequencing. Gene expression was compared for irradiated cells vs. unirradiated controls under normoxia (A and C) and hypoxia following X-rays (A and B, respectively) and ^12^C ions (C and D, respectively) exposure. Furthermore, the effects of oxygenation status on the response to X-rays (E) and ^12^C ions (F) exposure were determined by comparing respective irradiated samples. The radiation quality’s effect was evaluated by comparing the response to X-ray vs. ^12^C ion exposure under normoxia (G) and hypoxia (H). The top 15 GO terms were selected by *p*-value for each comparison. Color intensity represents the logarithm of the adjusted *p*-values, while gray means that the pathway was not significantly enriched in GSEA for this comparison.

Gene set enrichment analysis (GSEA) performed to evaluate A549 cell processes being enriched following irradiation under normoxia and hypoxia showed greater regulation of mitosis after X-ray exposure (Figure 5, columns A and B) compared to carbon ion exposure (Figure 5, columns C and D) under both normoxia and hypoxia.

Additionally, X-ray exposure under hypoxia (Figure 5, column B) enriched processes about locomotion, cell motility, and cell migration that were not enriched under normoxia (Figure 5, column A). Differentially expressed genes in irradiated cells compared to unirradiated controls were analyzed using standard lists of cell cycle-related genes available in the KEGG and Reactome databases [28,29] to identify cell cycle response genes that were differentially expressed following X-rays and carbon ion exposure under normoxia and hypoxia (Table A1). The general cell cycle response 4 h after irradiation appeared to be similar between normoxic and hypoxic cells regardless of radiation quality (X-rays vs. ^12^C ions) and was largely characterized by downregulation of genes regulating early and late mitosis. Additionally, expression of the p53-mediated cell cycle inhibitor *CDKN1A* was strongly upregulated after both X-rays and ^12^C exposure, independent of oxygenation status. This upregulation was accompanied by a counter-upregulation of the cell cycle promoter MDM2.

#### 2.4.2. Oxygenation Status Impacts Radiation Response in Terms of Extracellular Matrix, Cytoskeleton, and Chromatin Organization

GSEA showed that the primary enrichment of cell processes following irradiation under hypoxia compared to that under normoxia is similar for both X-rays and carbon ion exposure (Figure 5, columns E,F). This comprises extracellular matrix organization, cell migration, cell motility, and locomotion.

Gene expression response following irradiation under normoxia differed between X-rays and ^12^C ions (Figure 5, column G), mainly in cell processes about chromatin and chromosome organization. On the other hand, the hypoxia-modulated gene expression response differed between X-rays and carbon ions (Figure 5, column H) in cell processes governing cytoskeleton reorganization and positive regulation of cell communication and signaling.

#### 2.4.3. Radiation Response under Hypoxia Involves Upregulation of PI3K/AKT Target Genes

Analyzing genes constituting the radiation response in cell cycle regulation and cell migration/motility (Section 2.4.1 and Section 2.4.2) revealed many of them to be target genes of the PI3K/AKT pathway (Table A3 and Table A4).

PI3K/AKT pathway target genes (Table 3) involved in cell cycle processes were upregulated in response to radiation exposure generally irrespective of oxygenation status and radiation quality, while those related to processes of cell motility, migration and locomotion were found to be mainly differentially upregulated following irradiation under hypoxia or by hypoxia alone.

The PI3K/AKT pathway target genes activation signature following irradiation compared to unirradiated controls (Table 3) comprised of four genes regardless of oxygenation status and radiation quality: Cyclin-dependent kinase inhibitor 1A (*CDKN1A*), murine double minute 2 proto-oncogene (*MDM2*), placental growth factor (*PGF*) and KIT ligand (*KITLG*). While *CDKN1A* and *MDM2* regulate the cell cycle, *PGF* and *KITLG* are pleiotropic factors that promote cell proliferation and migration.

Prolonged hypoxia (48 h + 4 h) in the absence of irradiation produced upregulation of nine PI3K/AKT target genes in comparison to normoxia. The same genes were found to be differentially upregulated under hypoxia following irradiation independent of radiation quality. However, in general, X-ray exposure under hypoxia results in higher fold change upregulation of these genes compared to ^12^C ion exposure. These genes are all mitogenic and mainly control cell proliferation and migration (Table A1).

#### 2.4.4. Expression of DNA Repair and Apoptosis Genes following Irradiation Appears Unaffected by Oxygenation Status and Radiation Quality

DEGs in our study were compared to those listed in the KEGG database to evaluate differential expression of genes related to DNA repair, apoptosis and autophagy. Four hours after irradiation, cells showed upregulation of two DNA repair genes of the Nucleotide Excision Repair (NER) pathway independent of the radiation quality and the oxygenation status (Table A2), but genes related to Homologous repair (HR) and Nonhomologous end joining (NHEJ) were not found to be affected.

The FAS cell surface death receptor gene (*FAS*) involved in apoptosis’ extrinsic pathway was upregulated in response to irradiation independent of radiation quality and slightly enhanced under hypoxia in irradiated cells.

## 3. Discussion

Our experiments showed that A549 NSCLC cells required late plating (LP) to exhibit radioresistance under hypoxia (1% O_2_), as immediate plating (IP) of irradiated A549 cells led to greater radiosensitivity under hypoxia compared to normoxia (20% O_2_), regardless of the radiation dose (0–4 Gy) or quality (low LET X-rays vs. high LET ^12^C ions). We assessed radiosensitivity in terms of change in the clonogenic potential of irradiated cells using Puck’s colony forming ability assay, as it remains the gold standard for radiosensitivity evaluation studies [30,31].

Irradiated hypoxic cells, when plated immediately, might be more sensitive to the conditions of the CFA assay, i.e., a very low number of cells per culture vessel resulting in early cell death of some hypoxic irradiated cells and lower colony formation. In LP experiments, the cells had 24 h repair time in a confluent cell layer before reseeding, which might have supported survival of irradiated hypoxic cells. Furthermore, this effect of the plating time point on clonogenic cell survival under hypoxia was more pronounced following X-ray exposure than that of ^12^C ions. This was exemplified by an only ~20% increase in OER under hypoxia by switching from immediate to late plating in the case of ^12^C ion exposure in comparison to a doubling of OER for X-ray exposure (Table 1).

Survival fraction comparisons have been reported between high- and low-LET exposure in the A431, SQ20B and FaDu cell lines under normoxia and hypoxia, where hypoxia increases cell survival compared to normoxia following low-LET irradiation but not after high-LET irradiation [21,22]. These studies reported hypoxia-induced radioresistance despite immediate plating, as opposed to our study. This may be because hypoxic cells were returned to a normoxic environment after irradiation, which introduced reoxygenation as a confounding factor that probably suppressed the early death of some of the hypoxic cells, whereas our study examined radiotoxicity in A549 NSCLC cells maintained under continuous hypoxia before, during and after irradiation.

Under normoxic conditions, the RBE of ^12^C ions in the spread-out Bragg peak (LET 50–70 KeV/µm) is generally reported to be about 3 [32], which is comparable to our findings. The observation that the RBE of ^12^C ions under hypoxia remains comparable to that under normoxia (in late plate plating experiments) has been reported in the literature for A549 cells incubated at 1% O_2_ starting 16 h before irradiation with ^12^C ions in the spread-out Bragg peak [1]. In this work, the observed RBE decrease of ^12^C ions under hypoxia (IP CFA) was solely caused by the increased radiosensitivity of A549 cells in IP experiments under continuous hypoxia, as the D_0_ values of ^12^C ions under normoxia and hypoxia both amounted to 1.1 Gy. The higher RBE of ^12^C ions compared to X-rays with lower plating-based OER fluctuations suggests that high-LET particle radiation might be more efficient in NSCLC radiotherapy in killing hypoxic cells than conventional X-rays.

Cell cycle phases’ distribution was analyzed in A549 cells under hypoxia compared to normoxia in the presence and absence of radiation exposure (X-rays and ^12^C ions) because cell cycle arrest constitutes a vital part of the DNA Damage Response (DDR) to ionizing radiation. It occurs predominantly at the G2/M checkpoint but also at G1/S and mitotic checkpoints [33], providing time for DNA repair. As expected, prolonged and continuous hypoxia (1% O_2_ for ≥48 h) resulted in a slowing down of the overall proliferation rate, manifesting as a redistribution of hypoxic cells toward G1 and away from G2, as reported previously in the literature [16,17,18,34]. Hypoxia (1% O_2_) has been reported to increase the doubling time of A549 cells in vitro by 32% compared to normoxia (20% O_2_) [35]. After irradiation, the decline in the G1 population and the associated increase in the G2 population were much less pronounced under hypoxia than under normoxia. This stunting of the G2 cell cycle arrest under hypoxia was more obvious following exposure to X-rays compared to ^12^C ions. The findings might have clinical relevance, as a smaller G2 arrest results in a lower redistribution of tumor cells in the cell cycle, causing lower radiosensitivity at the next fraction during radiotherapy by resulting in a smaller fraction of cells in the radiosensitive cell cycle phases (i.e., late G2/M) [36]. A high dose of 8 Gy was used to best accentuate the effect of irradiation on the cell cycle, while keeping it relevant to doses that are in clinical use.

We studied DNA double-strand breaks (DSBs) induction and resolution under normoxia and hypoxia following irradiation (X-rays and ^12^C ions) as they are the most lethal ionizing radiation-induced damage with the lowest probability of error-free repair. Seeing no significant difference in DSB induction and resolution under normoxia and hypoxia following irradiation, along with a lower initial number of DSBs produced after ^12^C ion exposure compared to X-rays, is supported by a study reported by Wozny et al. who found no significant difference in γH2AX foci induction (30 min post-irradiation) or resolution (24 h post-irradiation) between normoxia and hypoxia in laryngeal squamous cell carcinoma SQ20B cells. They also reported fewer foci with ^12^C ions compared to X-ray exposure, whereby 2 Gy of X-rays resulted in 30.6 ± 1.7 foci, whereas 2 Gy of ^12^C ions produced 18 ± 1.7 foci 30 min after irradiation under normoxia [22]. This LET-based difference in DSB induction may be explained by the highly concentrated energy deposition within small tracks by ^12^C ions when high-LET radiation passes the cell compared to low-LET X-rays. Furthermore, the number of γH2AX foci counted in our experiments was close to the calculated hits (20 per cell nucleus) using Poisson’s statistics. A dose of 2 Gy was used to achieve a quantifiable number of foci, as at higher doses, counting individual foci was hampered by confluence of the fluorescent spots in the cell nucleus.

We carried out global gene expression analysis to identify molecular clues explaining the differences in cell survival and cell cycle modulation in hypoxic and normoxic A549 cells. The effect of hypoxia alone on the enrichment of cell migration and cell motility seen in our study has been reported previously in A549 cells incubated at 1% O_2_ for 12 h, for which AKT pathway activation was suggested as a potential mechanism [37]. X-ray irradiation under hypoxia compared to that under normoxia further enriched cell migration and cell motility processes, while no such enrichment was observed following carbon ion exposure under hypoxia compared to that under normoxia. Recently, exposure to ^12^C ions has been reported to reduce cell migration and motility in comparison to X-ray exposure in two different cancer stem cell lines [38]. The suggested reason is a more uniform ROS production following X-ray exposure, resulting in greater oxidative stress that acts as a trigger for increased cell motility and migration. This effect was suggested to be initiated by HIF-1α activation in response to irradiation with X-rays, which did not occur after ^12^C ion exposure of head and neck squamous cell carcinoma (HNSCC) cells [39]. In A549 cells, HIF-1α was even reduced after ^12^C ion exposure [32]. Cell migration and cell motility are functional endpoints of the epithelial-mesenchymal transition (EMT), which is a mechanism of radioresistance under hypoxia [40,41]. The GSEA findings may therefore hint at a possible EMT-based mechanism responsible for hypoxia-induced radioresistance to X-rays that is absent against ^12^C ions.

We studied DEG expression under normoxia and hypoxia following irradiation in light of the PI3K/AKT signaling pathway as it represents a link between cell survival and cell cycle response following radiation-induced DNA damage [42,43,44]. PI3K/AKT target genes found upregulated in our study under hypoxia in comparison to normoxia in the absence or presence of irradiation serve as good candidates for eliciting hypoxia-induced radioresistance, as they have been reported to be associated with treatment resistance and tumor aggressiveness through sustained cell proliferation, inhibition of cell death and increased cell migration/motility. Additionally, pyruvate-dependent kinase 1 (*PDK1*) upregulation under hypoxia may provide a mechanistic explanation for a slower proliferation rate under hypoxia and a greater overall G1 population, as it inactivates pyruvate dehydrogenase, which is needed to irreversibly produce pyruvate that is then channeled into the tricarboxylic acid (TCA) cycle. *PDK1* upregulation, therefore, metabolically shifts cells away from TCA and toward glycolysis, slowing down the cell cycle but providing survival advantages, such as glucose availability for nucleotide metabolism [35]. *PDK1* upregulation has been reported as a cause of treatment resistance in NSCLC [45].

The PI3K/AKT pathway also explains the cell cycle response to radiation in our study through strong *CDKN1A* upregulation seen in A549 cells independent of oxygenation status and radiation quality. While *CDKN1A* is primarily regulated by p53, the PI3K/AKT pathway has been reported to increase its expression by phosphorylating microphthalmia-associated transcription factor (*MITF*), which then binds to *TP53* and increases *CDKN1A* expression [46]. PI3K/AKT signaling has been demonstrated to increase *CDKN1A* expression in prostate cancer [47].

The absence of upregulation of DSB repair genes supports results from our γH2AX experiments that DSB repair kinetics were similar under both oxygen conditions following either X-ray exposure or carbon ion exposure, while higher expression of *FAS* following irradiation of hypoxic cells compared to normoxic cells, both for X-rays and ^12^C ions, may support our hypothesis of early cell death in CFA IP experiments under hypoxia.

## 4. Materials and Methods

### 4.1. Cell Cultivation

A549 cells (human, male, lung adenocarcinoma; KRAS mutated, p53 wildtype [48] were purchased from LGC Genomics (Berlin, Germany) and cultured in 25 cm^2^ or 80 cm^2^ cell culture flasks (Labsolute, Th. Geyer GmbH, Renningen, Germany) at a density of 5000 cells/cm^2^, using Alpha-Minimally Essential Medium (α-MEM; PAN Biotech, Aidenbach, Germany) containing 10% (*v*/*v*) dialyzed Fetal Bovine Serum (FBS; PAN Biotech), 2% (*v*/*v*) sterile glucose solution (0.94 mol/L), 1% (*v*/*v*) Penicillin (10,000 U/mL)/Streptomycin (10 mg/mL) (PAN Biotech), 1% (*v*/*v*) Neomycin/Bacitracin (Biochrom AG, Berlin, Germany), and 1% (*v*/*v*) Amphotericin (250 µg/mL) (PAN Biotech). For immunofluorescence experiments (Section 4.4), cells were cultured on glass coverslips (∅10 mm, Paul Marienfeld GmbH & Co. KG, Lauda Königshofen, Germany) placed in Petri dishes (∅3 cm) (Greiner Bio-One GmbH, Frickenhausen, Germany) and in 9 cm^2^ slide flasks (ThermoFisher Scientific, Waltham, MA, USA) using the same cell density and culture medium as described above.

The cells were incubated at 37 °C and saturated humidity either under normoxia (20% O_2_) in a CO_2_ incubator (5% CO_2_; Heraeus HERAcell 150, Thermo Fisher Scientific, Karlsruhe, Germany) or under hypoxia (1% O_2_) in an InvivO_2_ 400 hypoxia workstation (Baker Ruskinn, South Wales, UK) flushed with 5% CO_2_, 1% O_2_, and 94% N_2_. The incubation time in culture under normoxia or hypoxia before irradiation was 48 h to allow the cells to enter the exponential growth phase.

### 4.2. Irradiation

**Figure 6:**
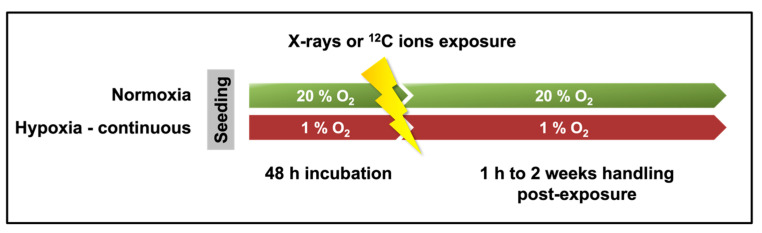
Cells were incubated under normoxia (20% O_2_) or hypoxia (1% O_2_) for 48 h, following which they were irradiated with X-rays or ^12^C ions. Subsequent experimentation until fixation of cells was also carried out under 20% O_2_ for normoxic cells and at 1% O_2_ for hypoxic cells.

After 48 h of incubation, the A549 cells were irradiated with either X-rays or ^12^C ions (Figure 6). The caps of the culture flasks were tightened before transferring them for irradiation. The flasks housing hypoxic cells were shifted for irradiation in air-tight boxes before exporting them out of the hypoxia workstation. They were only taken out from the air-tight boxes for the brief minutes of actual irradiation, following which they were returned into them for their transport back. Several oxygen readings were taken before actual experiments using the Seven2go dissolved oxygen meter S9 (Mettler Toledo, Giessen, Germany) to ensure that this method did not lead to a significant change in oxygen concentration within the medium of the flasks housing the hypoxic cells.

X-ray exposure (LET: 0.3–3.0 KeV/µm) was performed in an RS 225 X-ray chamber (X-strahl, Ratingen, Germany) at a stable dose rate of 1.0 Gy/min, which was ensured by keeping the distance of the sample from the X-ray source to 450 mm. Low-energy X-rays were eliminated using a copper (Cu) filter with a thickness of 0.5 mm. The dose and dose rate were monitored using the UNIDOS^webline^ dosimeter with the ionization chamber TM30013 (PTW, Freiburg, Germany).

**Figure 7:**
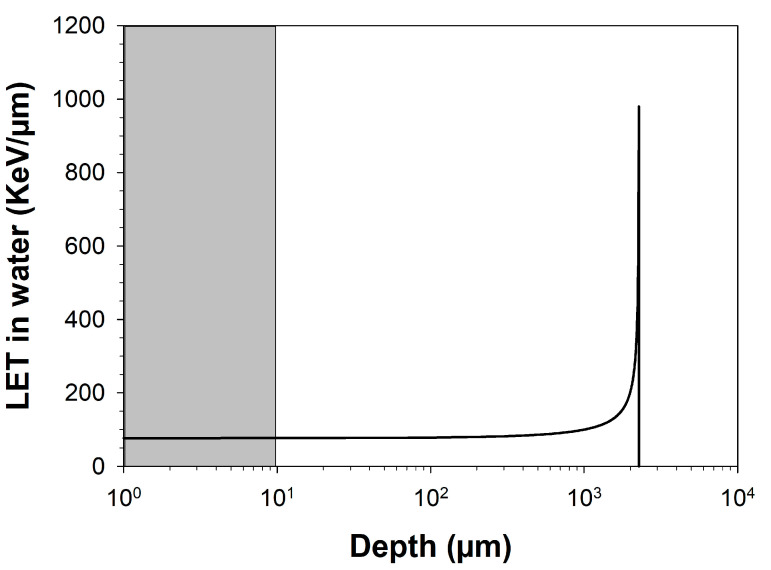
Bragg curve of ^12^C ions beam (25.7 MeV/n). It shows the linear energy transfer (LET) of carbon ions as they pass through a water phantom. The LET in water at different depths was calculated using “Energy vs. LET vs. Range calculator version 1.24”. The LET remained constant at ~75 KeV/µm to a depth of ~1000 µm beyond which there was a sharp surge to over 1000 KeV/µm, the Bragg peak, followed by a sharp drop to zero indicating the stopping of the ions. The cells (their estimated maximal thickness is represented in gray) were irradiated in the plateau phase of the curve at a constant LET of ~75 KeV/µm.

Carbon ion exposure was carried out at a heavy ion accelerator at a dose rate of 1 Gy/min. During carbon ion exposure, cells were placed in the plateau region of the Bragg curve, resulting in a constant LET over the thickness of the cells (Figure 7). To attain an LET (in water) relevant for clinical settings (~75 keV/µm), the carbon ion beam energy was reduced from 95 MeV/n to 35 MeV/n by placing a polymethyl methacrylate (PMMA, thickness 16.9 mm) energy degrader in the beam. The energy was further reduced by the polystyrene bottom of the cell culture flask, resulting in an energy of 25.7 MeV/n and a calculated LET in water of 73 keV/µm. The remaining range of ions in water was 2550 µm, indicating that the cells were exposed in the plateau region of the Bragg curve. The fluence for heavy ions (P/cm^2^) was used to calculate the radiation dose (Gy) (41).

The number of expected carbon ion hits to the cell nucleus was calculated based on an average cell nucleus area of 118.8 ± 52.5 µm^2^ using the Poisson distribution (Table 4). Since the cell culture flasks had to be kept upright during carbon ion exposure due to the horizontal beam setup, the flasks were filled to the neck with a culture medium to prevent desiccation of the cells during exposure.

Following irradiation, normoxic flasks underwent a medium change under the type 2 laminar flow hood and then returned to the CO_2_ incubator with loosened caps until subsequent experimentation. The hypoxic flasks underwent medium change inside the hypoxia workstation, where they then stayed with loosened caps until subsequent handling. The medium used for the purpose was degassed by warming it to 25 °C in the Sonorex Digiplus ultrasonic water bath (Bandelin, Berlin, Germany) for 40 min followed by placing it in the hypoxia workstation for another 40 min with a loosened bottle cap before use. All other liquids, such as trypsin or PBS, were degassed in the same way before use.

### 4.3. Cell Survival Analysis

Puck‘s colony forming ability (CFA) assay was performed to compare surviving cell fractions of A549 cells cultured under normoxia (20% O_2_) and hypoxia (1% O_2_) following different doses of X-rays and ^12^C ions (0.5, 1, 2 and 4 Gy).

The irradiated cells were trypsinized and seeded in petri dishes (∅6 cm LABsolute, Th. Geyer GmbH, Renningen, Germany), either immediately after irradiation (immediate plating) or after a delay of 24 h (late plating). The cell colonies, once visible, were fixed and stained with 5 mL of crystal violet–formaldehyde staining solution for 20 min after removing culture medium from the petri dishes. Stained colonies comprising over 50 cells were counted using a manual colony counter (Schuett count, Schuett-biotec, Göttingen, Germany). Survival curves were generated for each oxygen condition and radiation quality by plotting the surviving fractions on a logarithmic scale as a function of dose on a linear scale. The single-hit multi-target model was used to perform regression analysis of experimental data, and model parameters such as D_0_ and n were computed [49]. The Relative Biological Effectiveness (RBE) of ^12^C ions was calculated by Equation (1):

(1) $$\mathrm{RBE}\;\mathrm{Survival}\;\mathrm{reduction}=\frac{D_{0(X-rays)}}{D_{0(Testradiation)}}$$

The Oxygen Enhancement Ratio (OER) of hypoxia was calculated by Equation (2):

(2) $$\mathrm{OER}=\frac{D_{0(\mathrm{Hypoxia})}}{D_{0(\mathrm{Normoxia})}}$$

### 4.4. DNA Double-Strand Break Analysis

DNA double-strand breaks were analyzed through γH2AX immunofluorescence microscopy of cells grown under normoxia and hypoxia following irradiation with a 2 Gy dose of X-rays or ^12^C ions. The cells were fixed in 3.5% formaldehyde for 30 min at 4 °C at various time points (1, 2, 6, 12, 18 and 24 h) after irradiation. The fixed cells were permeabilized by adding a solution of 5% normal goat serum (NGS), 1% dimethyl sulfoxide (DMSO), and 0.3% Triton X-100 in PBS for 1 h at room temperature. They were then stained with the primary antibody, Alexa Fluor 488 Mouse anti-γH2AX clone 2F3 (Biolegend, San Diego, CA, USA), diluted (1:250) in a staining solution comprised of PBS with 1% DMSO and 0.3% Triton X-100, and incubated overnight at 4 °C. The next day, after washing three times with PBS, cells were stained with the secondary antibody goat anti-mouse IgG-Atto488 (1:1000, Sigma Aldrich, Saint Louis, MO, USA) and the nuclear stain 4′,6-diamidino-2-phenylindole (DAPI) (0.5 µg/mL stock solution diluted at 1:400), followed by an incubation of 45 min in the dark at room temperature before slide preparation.

Microscopy was carried out using a Zeiss Axio Imager M2 (Carl Zeiss NTS GmbH, Oberkochen, Germany). Eighteen images per cover slip were taken using the DAPI and Atto488 channels, keeping exposure time constant across each biological replicate. The number of γH2AX foci within each cell nucleus was counted using Image J software version 1.53 (NIH, Bethesda, MD, USA) [50].

### 4.5. Analysis of Cell Cycle Response

**Figure A1:**
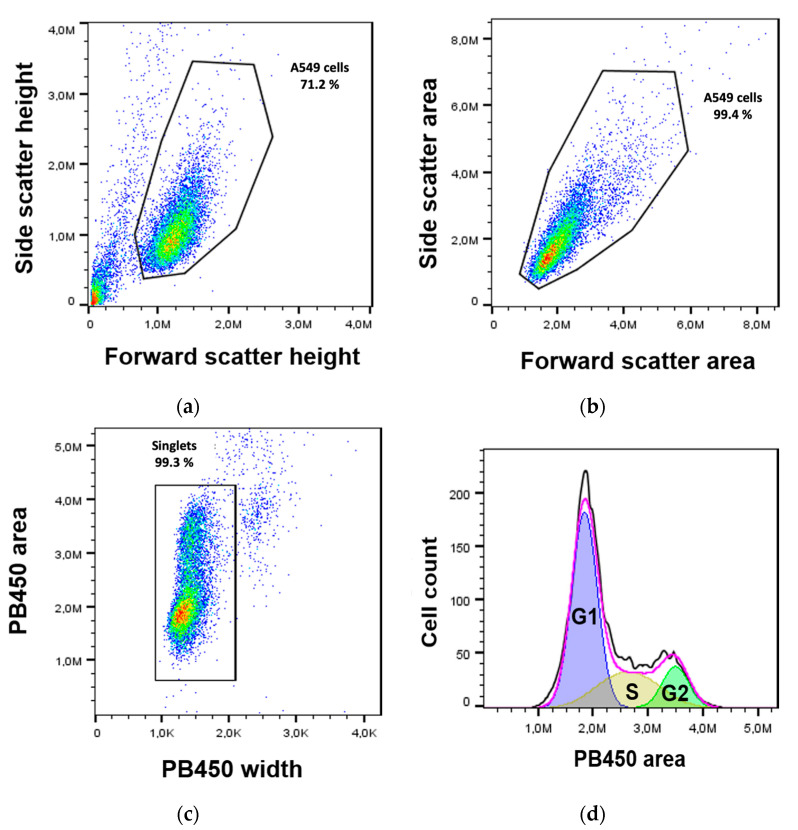
Gating strategy for cell cycle analysis. A549 cells were separated from cell debris by applying appropriate gates on forward and side scatter height (**a**) and area (**b**) signal plots. A549 singlets were separated based on DNA content through gating of the PB450 channel signal area vs. width plot (**c**) where the PB450 channel allows the measurement of the DAPI emission signal. Finally, cell cycle phase distribution was obtained by plotting cell count as a function of the PB450 channel area signal of all cells in the singlets gate (**d**).

Following irradiation of normoxic and hypoxic cells with 8 Gy of X-rays or ^12^C ions, they were fixed in 3.5% formaldehyde at defined time points (2, 6, 12, 18, and 24 h after irradiation) after detaching them with 1 mL Trypsin/EDTA solution. Thirty minutes after fixation, the cells were washed in PBS, and nuclei were stained with freshly prepared DAPI (500 ng/mL) and Triton X-100 (3 µg/mL) solution in PBS and kept in the dark for 30 min at room temperature. The nuclear DNA content of the cells was measured by flow cytometry (Cytoflex S, Beckman Coulter, Indianapolis, IN, USA) to determine their cell cycle phase distribution (Figure A1) using the Dean-Jett-Fox cell cycle mathematical model available in FlowJo software version 10 (BD Biosciences, San Jose, CA, USA) [51].

### 4.6. Gene Expression Analysis

To determine the global transcription profile of cells irradiated under normoxia and hypoxia with 8 Gy of X-rays or ^12^C ions, the culture medium was removed 4 h after irradiation, and cells were lysed using RLT buffer (Qiagen, Hilden, Germany) containing β-mercaptoethanol (1:100, Sigma Aldrich) and RNA was isolated with RNeasy Mini Kit (Qiagen). RNA concentration and integrity were determined using the RNA 6000 Nano Assay (Agilent Technologies, Böblingen, Germany). Three micrograms of total RNA per sample (4 biological replicates per condition) were sent on dry ice to GENEWIZ (Leipzig, Germany) for mRNA sequencing in the same run after Poly(A) selection using the Illumina NovaSeq6000 platform (configuration: 2 × 150 bp, 350M read pairs). GENEWIZ mapped the reads onto the *Homo sapiens* GRCh38 reference genome and calculated unique gene hit counts falling within exon regions. We then employed the DESeq2 package in R [52] for differential gene expression analysis and used the expression data to perform the Gene Set Enrichment Analysis (GSEA) [53]. Genes with an adjusted *p*-value < 0.05 and absolute log_2_ fold change >1 were considered differentially expressed genes for each group comparison.

### 4.7. Statistical Analysis

Three independent biological experiments with multiple technical replicates for each experimental condition were conducted except for RNA sequencing, where 4 biological replicates were performed. Basic data handling was performed using Excel software version 2016 (Microsoft corporation, Redmond, WA, USA). For graphs and significance testing, GraphPad Prism 9 (Dotmatics, Boston, MA, USA) was used except for CFA data, where curves were plotted using Sigma Plot 15 (Systat Software Inc., Palo Alto, CA, USA). Two-way ANOVA for testing cell cycle and γH2AX data, multiple two-way unpaired *t*-tests to evaluate CFA data, while the Wald test was used to calculate *p*-values and the Benjamini-Hochberg test for calculation of adjusted *p*-values (padj) in case of RNA sequencing data.

## 5. Conclusions

Carbon ion irradiation might be a better treatment option for overcoming hypoxia-induced radioresistance in non-small cell lung carcinoma than X-rays or other low-LET radiation on account of higher RBE and more stable OER.

Gene expression analysis highlights potential therapeutic targets that influence cell migration and motility and are upregulated under hypoxia and to a greater extent after X-ray exposure. This may be of clinical significance, as their inhibitors, when used as radiosensitizers, may improve the cytotoxicity of X-rays against hypoxic NSCLC cells. Using a dose of 8 Gy to evaluate gene expression response provides an opportunity to examine our findings in the context of gene expression in response to stereotactic body radiotherapy, which employs high doses per fraction to treat NSCLC. Similarly, with the recent increase in heavy ion particle therapy trials, our results may provide useful insights to researchers working on the hypoxic and oxic response of NSCLC to clinical-range LET ^12^C ions, in terms of cell reproductive integrity, cell cycle response and gene expression.

However, to generalize the findings to NSCLC, the investigation has to be expanded to other human NSCLC cell lines. Genes of interest commonly upregulated on mRNA and protein levels across multiple NSCLC cell lines under hypoxia may then be shortlisted for knockout or knockdown studies to validate their impact on hypoxia-induced radioresistance in NSCLC in response to low- and high-LET radiation.

## Figures and Tables

**Table 1:** Parameters of the survival curves for A549 cells following exposure to X-rays or carbon ions under normoxia and hypoxia.

Radiation Quality	Assay Type	Oxygen Protocol	D_0_ * µ ± SD	*n* * µ ± SD	OER * µ ± SD	RBE * µ ± SD
X-rays	Immediate Plating	Normoxia	2.98 ± 0.20	1.07 ± 0.05	0.57 ± 0.04	1 by definition
		Hypoxia	1.68 ± 0.08	1.20 ± 0.07		
	Late Plating	Normoxia	2.29 ± 0.13	0.94 ± 0.05	1.10 ± 0.08	
		Hypoxia	2.50 ± 0.16	1.19 ± 0.06		
^12^C ions	Immediate Plating	Normoxia	1.11 ± 0.07	0.64 ± 0.08	1.00 ± 0.10	2.68 ± 0.47
		Hypoxia	1.10 ± 0.11	0.34 ± 0.06		1.52 ± 0.11
	Late Plating	Normoxia	0.90 ± 0.03	0.76 ± 0.07	1.19 ± 0.10	2.54 ± 0.81
		Hypoxia	1.08 ± 0.10	0.49 ± 0.09		2.33 ± 0.29

* “D_0_” is the dose that reduces the surviving cells to a fraction of 37% of the original cell number before the radiation dose in the exponential part of the survival curve. “*n*” is a hypothetical number along the y-axis of the cell survival graph derived by extrapolating the straight part of the cell survival curve up to the y-axis. OER, oxygen enhancement ratio; RBE, relative biological effectiveness of ^12^C ions compared to X-rays under either normoxia or hypoxia (1% O_2_).

**Table 2:** Average distribution of A549 cells in the cell cycle phases over 24 h ^†^ following an incubation of 48 h under hypoxia or normoxia.

Cell Cycle Phase	Cell Population (%) Over 24 h in Absence of Irradiation (µ ± SE)		Statistical Significance (α < 0.05)
	O_2_ Protocol		
	Normoxia	Hypoxia	
G1	59.64 ± 0.77	67.29 ± 1.92	*
S	17.25 ± 2.55	17.66 ± 1.51	ns
G2	23.59 ± 2.15	16.67 ± 1.19	*

^†^ Cell cycle phase distribution was determined 2, 6, 12, 18 and 24 h after the 48 h preincubation under hypoxia. Ns: not significant; *: *p* < 0.05; n = 6.

**Table 3:** Differentially expressed PI3K/AKT target genes after exposure to X-rays or ^12^C ions under normoxia and hypoxia.

DEGs (Gene Name Abbreviation)	Gene Expression Based on *p*-Adjusted log2 Fold Changes						
	Irradiated vs. Unirradiated				Hypoxic vs. Normoxic		
	X-rays		^12^C Ions		Controls (H0 vs. N0)	X-rays (H8 vs. N8)	^12^C ions (H8 vs. N8)
	Normoxia (N8 ^a^ vs. N0)	Hypoxia (H8 vs. H0)	Normoxia (N8 vs. N0)	Hypoxia (H8 vs. H0)			
*CDKN1A*	**2.63 ^b^**	**2.62**	**2.61**	**2.38**	−0.19	−0.21	−0.42
*MDM2*	**2.47**	**2.65**	**2.14**	**2.16**	−0.44	−0.26	−0.43
*PGF*	**2.46**	**2.65**	**2.59**	**3.05**	−0.56	−0.37	−0.10
*KITLG*	1.01	**1.38**	**1.36**	**1.46**	−1.10	−0.73	−1.00
*SYK*	**1.40**	1.06	**1.58**	1.26	−0.07	−0.41	−0.39
*COL9A2*	1.24	**1.68**	0.92	**1.72**	0.13	0.56	0.93
*ANGPT4*	0.90	1.17	1.88	1.36	0.27	0.55	−0.25
*PDGFRB*	0.90	1.21	1.04	**1.96**	−0.12	0.19	0.80
*GDNF*	**2.29**	1.39	0.91	−0.34	0.88	−0.02	−0.37
*PDK1*	0.05	−0.24	−0.42	−0.16	**1.90**	**1.62**	**2.16**
*FGF1*	0.24	0.71	0.18	0.67	**1.36**	**1.84**	**1.85**
*DDIT4*	0.53	−0.27	0.33	−0.07	**1.75**	**0.95**	**1.35**
*ITGB6*	−0.03	0.08	−0.24	0.07	**1.35**	**1.46**	**1.66**
*COL1A1*	0.05	-0.01	−0.36	0.20	**1.80**	**1.75**	**2.36**
*PDGFB*	−0.17	0.15	−0.21	−0.08	**1.63**	**1.95**	**1.75**
*LAMC2*	−0.21	0.31	−0.05	−0.28	**1.64**	**2.16**	**1.42**
*EFNA3*	−0.62	−0.62	−0.47	−0.52	**1.44**	**1.44**	**1.39**
*LPAR5*	0.33	0.72	−0.07	0.34	**2.39**	**2.79**	**2.81**
*GNG4*	0.50	0.27	−0.28	0.26	0.85	0.63	**1.39**
*EFNA1*	0.09	−0.02	−0.36	0.87	0.75	0.64	**1.98**
*ITGA2*	0.00	0.71	0.43	0.23	0.89	**1.61**	0.69

**^a^** N8, A549 cells exposed to 8 Gy X-rays or ^12^C ions under normoxia. N0, A549 cells exposed to 0 Gy X-rays or ^12^C ions under normoxia. H8, A549 cells exposed to 8 Gy X-rays or ^12^C ions under hypoxia. H0, A549 cells exposed to 0 Gy X-rays or ^12^C ions under hypoxia. **^b^** Significant *p*-adjusted log2 fold changes of 1.33 (actual fold change of 2.5) or more are given in bold. DEGs, differentially expressed genes.

**Table 4:** Calculation of carbon ion hits to the cell nucleus of A549 cells for exposure with ^12^C ions with an energy of 25.7 MeV/n on target and an LET of 73 keV/µm.

Fluence (P/cm^2^)	Dose (Gy)	Unhit Fraction	Hit Fraction	Average Hits *
4.40 × 10^6^	0.5	0.01	0.99	5.1 (2.8–7.3)
8.79 × 10^6^	1.0	0.00	1.00	10.2 (5.7–14.7)
1.76 × 10^7^	2.0	0.00	1.00	20.3 (11.3–29.3)
3.52 × 10^7^	4.0	0.00	1.00	40.6 (22.7–58.6)
7.03 × 10^7^	8.0	0.00	1.00	81.3 (45.4–117.3)

* Values in parentheses indicate the average number of hits for cell nuclei that are one standard deviation smaller (66.3 µm^2^) or larger (171.4 µm^2^) than the average (118.8 µm^2^).

## Data Availability

Research data are stored in an institutional repository and will be shared upon request to the corresponding author.

## Appendix B

**Table A1:** Expression of genes involved in cell cycle that were differentially regulated in hypoxia in comparison to normoxia 4 h after X-ray exposure of A549 cells.

Regulated DEGs (Gene Name Abbreviation)	Gene Expression Based on *p*-Adjusted log2 Fold Changes						
	Irradiated vs. Unirradiated				Hypoxic vs. Normoxic		
	X-rays		^12^C ions		Controls (H0 vs. N0)	X-rays (H8 vs. N8)	^12^C ions (H8 vs. N8)
	Normoxia (N8 ^a^ vs. N0)	Hypoxia (H8 vs. H0)	Normoxia (N8 vs. N0)	Hypoxia (H8 vs. H0)			
*CDKN1A*	**2.63 ^b^**	**2.62**	**2.61**	**2.38**	−0.19	−0.21	−0.42
*MDM2*	**2.47**	**2.65**	**2.14**	**2.16**	−0.44	−0.26	−0.43
*PLK1*	**−1.73**	**−2.01**	**−1.59**	**−1.61**	0.04	−0.24	0.02
*PIF1*	**−3.96**	**−3.50**	**−2.05**	**−1.99**	−0.08	0.37	−0.02
*CENPE*	**−1.71**	**−1.45**	−1.18	−1.17	0.19	0.45	0.20
*BORA*	**−1.59**	**−1.54**	−1.07	-0.86	0.09	0.13	0.30
*AURKA*	**−1.98**	**−1.82**	−1.30	−1.24	0.06	0.22	0.12
*HMMR*	**−1.48**	**−1.53**	−1.25	−1.10	0.09	0.04	0.25
*KIF20A*	**−1.51**	**−1.81**	**−1.41**	**−1.46**	0.14	−0.16	0.09
*CENPA*	**−1.86**	**−1.57**	−1.19	−1.04	0.17	0.46	0.32
*KIF18A*	**−1.30**	**−1.44**	−0.99	−0.77	−0.02	−0.17	0.19
*HJURP*	−1.21	**−1.34**	−0.65	−0.63	−0.25	−0.38	−0.23
*NDC80*	−1.14	**−1.35**	−0.82	−0.69	0.18	−0.03	0.31
*TUBB8*	**2.25**	0.49	**2.09**	**1.45**	#N/A ^c^	−0.74	#N/A
*CABLES1*	−0.35	−0.73	−0.15	−0.40	−1.00	**−1.38**	−1.25

^a^ N8, A549 cells exposed to 8 Gy X-rays or ^12^C under normoxia. N0, A549 cells exposed to 0 Gy X-rays or ^12^C under normoxia. H8, A549 cells exposed to 8 Gy X-rays or ^12^C under hypoxia. H0, A549 cells exposed to 0 Gy X-rays or ^12^C under hypoxia. ^b^ Significant p-adjusted log2fold changes of 1.33 (actual fold change of 2.5) or more are given in bold. ^c^ #N/A mRNA of this gene was not detected. DEGs, differentially expressed genes.

**Table A2:** Expression of genes involved in DNA repair and apoptosis that were differentially regulated in hypoxia in comparison to normoxia 4 h after X-ray exposure of A549 cells.

Regulated DEGs (Gene Name Abbreviation)		Gene Expression Based on *p*-Adjusted log2 Fold Changes						
		Irradiated vs. Unirradiated				Hypoxic vs. Normoxic		
		X-rays		^12^C Ions		Controls (H0 vs. N0)	X-rays (H8 vs. N8)	^12^C ions (H8 vs. N8)
		Normoxia (N8 ^a^ vs. N0)	Hypoxia (H8 vs. H0)	Normoxia (N8 vs. N0)	Hypoxia (H8 vs. H0)			
DNA repair	*XPC*	**1.47 ^b^**	**1.51**	**1.41**	**1.31**	−0.04	0.00	−0.14
	*POLH*	**1.70**	**1.67**	**1.67**	**1.58**	0.05	0.02	−0.05
	*DDB2*	**1.32**	0.97	**1.23**	**1.26**	0.15	−0.20	0.18
Apoptosis/ Inflammation	*FAS*	**2.06**	**2.75**	**2.31**	**2.85**	−0.71	−0.02	−0.17
	*IL1A*	**1.37**	**1.54**	0.43	1.26	**2.63**	**2.81**	**3.46**
	*IL1B*	0.56	**1.33**	**1.94**	0.19	**1.80**	**2.58**	0.05
	*IRAK3*	**1.60**	**1.55**	−0.08	1.53	0.16	0.12	**1.76**

**^a^** N8, A549 cells exposed to 8 Gy X-rays or ^12^C ions under normoxia. N0, A549 cells exposed to 0 Gy X-rays or ^12^C ions under normoxia. H8, A549 cells exposed to 8 Gy X-rays or ^12^C ions under hypoxia. H0, A549 cells exposed to 0 Gy X-rays or ^12^C ions under hypoxia. **^b^** Significant *p*-adjusted log2 fold changes of 1.33 (actual fold change of 2.5) or more are given in bold. DEGs, differentially expressed genes.

**Table A3:** Differentially upregulated PI3K/AKT target genes in hypoxic A549 cells in the absence (H0 ^a^ vs. N0) or presence (H8 vs. N8) of X-ray or ^12^C ion exposure.

Regulated DEGs (Gene Name Abbreviation)	Gene Function and Interpretation of Results
*PDK1*	Pyruvate dependent kinase 1 (*PDK1*) upregulation inactivates pyruvate dehydrogenase which is needed to irreversibly produce pyruvate that then is channeled into the tricarboxylic acid (TCA) cycle. *PDK1* upregulation, therefore, metabolically shifts cells away from TCA and toward glycolysis, slowing down the cell cycle but providing survival advantages such as glucose availability for nucleotide metabolism [35]. *PDK1* upregulation has already been reported as a cause for treatment resistance in NSCLC [45]. Its upregulation under hypoxia in our experiments therefore may provide a mechanistic explanation for slower proliferation under hypoxia and greater overall G1 population.
*DDIT4*	The overexpression of DNA damage inducible transcript 4 (*DDIT4*) is considered to inhibit autophagy by inhibiting mTORC1 in a variety of malignancies including NSCLC [54]. Its upregulation under hypoxia in our experiments may be a contributing factor in hypoxia-induced radioresistance.
*LPAR5*	*LPAR5* has recently been reported to be overexpressed in NSCLC [55] and knocking it out in A549 cells has been shown to inhibit apoptosis and oppose growth arrest following irradiation [56]. LPAR5 upregulation, therefore, offers a credible mechanistic justification for increased survival and less pronounced G2 arrest seen after irradiation of A549 cells under hypoxia in our experiments.
*PDGFB*	Platelet derived growth factor beta (*PDGFB*), fibroblast growth factor 1 (*FGF1*), laminin subunit gamma 2 (*LAMC2*), integrin beta subunit 6 (*ITGB6*) and Ephrin A3 (*EFNA3*) encode respective receptors involved in cell—extracellular matrix (ECM) signaling, which plays a positive role in cancer initiation and progression mainly through induction of EMT transcription factors that increase cell motility and promote cell migration [57,58,59,60,61,62,63].
*FGF1*	
*LAMC2*	
*ITGB6*	
*EFNA3*	
*COL1A1*	Collagen type 1 alpha 1 chain (*COL1A1*) upregulation under hypoxia provides further evidence for hypoxia induced EMT in A549 cells in our experiments. As a collagen that is expressed by mesenchymal cells, *COL1A1* expression is associated with EMT and its upregulation in response to hypoxia in NSCLC has also been reported [64].

**^a^** N8, A549 cells exposed to 8 Gy X-rays or ^12^C ions under normoxia. N0, A549 cells exposed to 0 Gy X-rays or ^12^C ions under normoxia. H8, A549 cells exposed to 8 Gy X-rays or ^12^C ions under hypoxia. H0, A549 cells exposed to 0 Gy X-rays or ^12^C ions under hypoxia. DEGs, differentially expressed genes.

**Table A4:** Differentially upregulated PI3K/AKT target genes in A549 cells following irradiation under normoxia (N8 ^a^ vs. N0) and hypoxia (H8 vs. H0).

Regulated DEGs (Gene Name Abbreviation)	Gene Function and Interpretation of Results
*CDKN1A*	The p53-mediated Cyclin dependent kinase inhibitor A (*CDKN1A*) is the dominant gene responsible for inducing cell cycle arrest in response to irradiation [65] and such appeared to be the case in our A549 irradiation experiments, independent of oxygenation status.
*MDM2*	Mouse double minute 2 homolog (*MDM2*) is the chief negative regulator of p53 and its overexpression following irradiation is regarded as a homeostatic compensation to p53 activation [66].
*PGF*	Placental growth factor (*PGF*) is an angiogenic growth factor. Its overexpression of *PGF* has been associated with treatment resistance in ovarian cancer [67]. Its upregulation in our experiments following irradiation with X-rays and ^12^C ions suggests that it may be a cause for radioresistance in NSCLC independent of oxygenation status.
*KITLG*	KIT proto-oncogene ligand (*KITLG*) supports cell survival in a variety of tumors [68] and its overexpression is already documented in NSCLC [69]. Upregulation of *KITLG* has been associated with treatment resistance in nasopharyngeal cancer [68]. Its upregulation in our experiments following irradiation with X-rays and ^12^C ions suggests that it may be a cause for radioresistance in NSCLC independent of oxygenation status.
*COL19A2*	Irradiation upregulated Collagen type 9 alpha 2 chain (*COL9A2*) expression in A549 cells under hypoxia but not under normoxia. *COL9A2* is known to be a hypoxia-inducible gene [70], and its upregulation following irradiation under hypoxia may indicate greater risk of radiation fibrosis under hypoxia [71].

**^a^** N8, A549 cells exposed to 8 Gy X-rays or ^12^C ions under normoxia. N0, A549 cells exposed to 0 Gy X-rays or ^12^C ions under normoxia. H8, A549 cells exposed to 8 Gy X-rays or ^12^C ions under hypoxia. H0, A549 cells exposed to 0 Gy X-rays or ^12^C ions under hypoxia. DEGs, differentially expressed genes.
